# Two novel species of *Sympoventuriaceae* from palms in China

**DOI:** 10.3897/mycokeys.138.200127

**Published:** 2026-07-30

**Authors:** Qiu-Yue Zhang, Yu-Han Cao, Hui Sun, Lin Zhou, Lin Huang, Yun-Hong Tan, De-Wei Li, Li-Hua Zhu

**Affiliations:** 1 College of Forestry and Grassland, Nanjing Forestry University, Nanjing 210037, China Xishuangbanna Tropical Botanical Garden, Chinese Academy of Sciences Mengla China; 2 Collaborative Innovation Center of Sustainable Forestry in Southern China, Nanjing, 210037, China College of Forestry and Grassland, Nanjing Forestry University Nanjing China; 3 Xishuangbanna Tropical Botanical Garden, Chinese Academy of Sciences, Mengla, Yunnan 666303, China Collaborative Innovation Center of Sustainable Forestry in Southern China Nanjing China; 4 The Connecticut Agricultural Experiment Station Valley Laboratory, Windsor, CT 06095, USA The Connecticut Agricultural Experiment Station Valley Laboratory Windsor United States of America

**Keywords:** Fungal diversity, palm, phylogeny, *
Sympoventuriaceae
*, taxonomy

## Abstract

Palm plants (*Arecaceae*) are widely distributed ornamental hosts in southern China. However, most of these plants are introduced species, and their associated microfungal diversity remains poorly understood. In this study, four strains with morphological affinities to *Scolecobasidium* and *Yunnanomyces (Sympoventuriaceae)* were isolated from palm plants in Yunnan Province, China. Two novel species, *Scolecobasidium yunnanense* and *Yunnanomyces didymosporus*, are proposed based on morphological observations and separate multi-locus phylogenetic analyses: the phylogeny of *Scolecobasidium* was constructed from a concatenated ITS + nLSU + *tub2* + *tef1*-*α* dataset, while analyses for *Yunnanomyces* within *Sympoventuriaceae* relied on a combined ITS + nLSU + nrSSU + RPB2 dataset. *Scolecobasidium yunnanense* is characterized by its branched, septate, thick-walled pale hyphae, erect, unbranched and septate conidiophores often reduced to denticulate conidiogenous cells, and smooth, mostly 2-septate, ellipsoidal to fusiform conidia, measuring 8.3–12.6 × 2.0–3.5 μm. *Yunnanomyces didymosporus* is characterized by its monilioid mature hyphae, intercalary conidiogenous cells, and chain-forming mostly 1-septate conidia with a dark hilum on one or both ends, measuring 6.1–11.9 × 2.0–4.2 μm.

The study confirms their distinctiveness from extant species within each genus and enhances the understanding of microfungal diversity associated with palms.

## Introduction

Palm plants (*Arecaceae*) are defined by their large, typically palmately lobed or pinnately compound leaves, with ca. 2,600 recognized species globally (Dransfield et al. 2005; Dransfield et al. 2008). It has been estimated that around 76,000 fungal species are associated with palm hosts across the globe, yet only a little more than 2,500 of these fungi have been taxonomically described to date (Pereira and Phillips 2023). As economically important ornamental plants, palms are widely cultivated across southern China, yet over 80% of these cultivated palm taxa are exotic introductions. Moreover, frequent outbreaks of fungal pathogens severely impair the ornamental quality of cultivated palms (Peng et al. 2015; Zhou et al. 2025).

The genus *Scolecobasidium* E.V. Abbott (*Sympoventuriaceae*, *Venturiales*) was validly established by Abbott in 1927, to accommodate *S. constrictum* E.V. Abbott and *S. terreum* E.V. Abbott isolated from cotton and sugarcane soils in Louisiana, USA, with *S. terreum* designated as its type species (Abbott 1927). Members of *Scolecobasidium* are characterized by reduced, unbranched or rarely branched, brownish conidiophores, monoblastic or sympodial, denticle conidiogenous cells, and dark, didymo-, phragmo, or stauro, single, rhexolytic conidia; sexual morph is only known in *S. sexuale* (Samerp.et sl.) Crous, M. Shen and Y. Zhang ter, absent in other all species (Abbott 1927; Barron and Busch 1962; Seifert et al. 2011; Samerpitak et al. 2013; Song et al. 2023). The genus has a complex taxonomic history. Originally, *Scolecobasidium* was distinguished from other genera by the shape of its conidia and their arrangement on conidiophores (Abbott 1927). Later, more species with unbranched conidia were described, leading taxonomists to introduce the genus *Ochroconis* to accommodate these taxa (Samerpitak et al. 2014, 2017). Recently, with the development of molecular phylogenetics, most mycologists have accepted *Ochroconis* as a synonym of *Scolecobasidium*, and proposal of numerous new combinations (Hao et al. 2013; Shen et al. 2020; Wei et al. 2022). Recent phylogenetic analyses based on multiple loci (ITS, nLSU, *tef1*-*α*, and *tub2*) on *Scolecobasidium* clarified species through integrated morphological and multi-locus DNA data (Ren et al. 2013; Song et al. 2023). The genus is cosmopolitan, inhabiting diverse ecosystems such as soil, water, air, and plant tissues, including as saprobes or pathogens on plants and cold-blooded vertebrates (Shen et al. 2020). In addition, *Scolecobasidium* is the largest genus in the family *Sympoventuriaceae*, with over 100 records currently listed in Index Fungorum. Among the records, ca. 60 species were accepted in the genus (Hyde et al. 2024).

The genus *Yunnanomyces* Tibpromma & K.D. Hyde (*Sympoventuriaceae*, *Venturiales*) was established by Tibpromma et al. in 2018, typified with *Y. pandanicola* Tibpromma & K.D. Hyde (Tibpromma et al. 2018). It is distinguished by mononematous, fasciculate, branched or unbranched and hyaline to subhyaline conidiophores, holoblastic, monoblastic, integrated, terminal, determinate, cylindrical, and hyaline to subhyaline conidiogenous cells, and subglobose to ellipsoidal with solitary or arranged in a small chain, or muriform conidia; sexual morph absent in all species currently accepted (Tibpromma et al. 2018; Zhang et al. 2019; Barreto et al. 2025). Following its establishment, taxonomists have reported additional species from different regions, including China, Panama, South Africa, and Thailand, expanding the genus to encompass multiple taxa, which were primarily isolated from diseased leaves or dead rachides of monocotyledonous plants, such as *Arecaceae*, *Pandanaceae*, *Ruscaceae*, and *Sapotaceae* (Zhang et al. 2019; Crous et al. 2024; Liu et al. 2024; Zhang et al. 2024a, 2024b). Currently, Index Fungorum records nine species of *Yunnanomyces*, namely *Y. hagahagaensis* Crous, *Y. mangrovei* S.N. Zhang et al., *Y. muriformis* Y.R. Sun et al., *Y. narathiwatensis* O. Karimi et al., *Y. panamaensis* Crous, *Y. pandanicola*, *Y. phoenicis* S.N. Zhang & Jian K. Liu, *Y. sexualis* Zhi Yuan Zhang et al., *Y. syagri* G.G. Barreto et al. (Tibpromma et al. 2018; Zhang et al. 2019; Crous et al. 2024; Liu et al. 2024; Barreto et al. 2025; Karimi et al. 2025). Phylogenetically, previous studies consistently support *Yunnanomyces* as a monophyletic clade within the family *Sympoventuriaceae* based on multi-locus data (Crous et al. 2024; Liu et al. 2024; Barreto et al. 2025).

During a survey of microfungi on this palm species, four interesting fungal strains were collected, and exhibited morphological characteristics related to the anamorphic morphs of *Scolecobasidium* and *Yunnanomyces*. The fungi were subsequently isolated. Based on a combined analysis of morphology and multigene phylogeny, two new species were introduced to accommodate them. Furthermore, this study employed multilocus phylogenetic analyses and morphological studies to confirm their distinctions from existing species within their respective genera.

## Materials and methods

### Isolation of the potential fungal pathogen

Field surveys and sample collection were carried out in November 2024 at the Xishuangbanna Tropical Botanical Garden in Yunnan Province, China. The samples were taken specifically from senescent fronds, including leaves and petioles. After collection, the leaves were rinsed under tap water for 8–12 hours and then air-dried in a super clean bench over absorbent paper. Once the leaf surfaces were dry, the leaves were placed into the Petri dishes (150 mm) lined with filter paper. Sterile water was added to moisten the filter paper, and the dishes were sealed with parafilm, leaving a 1–2 cm gap for ventilation. These were then transferred to a climate chamber maintained at 25 ± 2 °C and 75% relative humidity, a method also employed by Zhou et al. (2025). Samples were inspected every three days for the formation of mycelia or fruiting bodies, maintaining moisture by adding sterile water as needed. Small number of mycelia or individual fruiting bodies were picked up with a loop and inoculated onto potato dextrose agar (PDA) plates, or the tip of the conidial pile was touched and streaked using the three-zone streaking method to isolate single spore colonies, thereby obtaining pure cultures. Pure cultures were grown on PDA at 25 ± 2 °C for 1–2 weeks.

All isolates are stored in the Forest Pathology Laboratory at Nanjing Forestry University. The holotype specimen of the new species is a living specimen being maintained via lyophilisation at the China Forestry Culture Collection Center (CFCC, https://cfcc.caf.ac.cn/), Chinese Academy of Forestry, Beijing, China, and the living ex-type cultures are maintained at the Forest Pathology Laboratory, Nanjing Forestry University.

### Morphological studies

Fungal cultivation and morphological characterization followed the protocols described by Zhou et al. (2025); specifically, the slide culture method was employed to ensure clear observation. Briefly, a 5-mm mycelial plug was placed centrally on PDA and incubated at 25 ± 2 °C in darkness. Colony features were recorded after 7 days. Microscopic observations were performed using Zeiss stereo and fluorescence microscopes, with structures mounted in distilled water. Morphological observations of reproductive structures were carried out using a compound microscope (Zeiss imager 2) with DIC and a Zeiss camera. Images were processed with ZEN 3.9 and Adobe Photoshop 2021. Sixty measurements were obtained per morphological structure, and colors were determined using Kornerup and Wanscher (1978).

### DNA extraction and sequencing

A cetyltrimethylammonium bromide (CTAB) rapid plant genome extraction kit (Aidlab Biotechnologies, Co., Ltd., Beijing, China) was used to extract DNA (Zhang et al. 2026). The nuclear ribosomal internal transcribed spacer (ITS), nuclear ribosomal large subunit (nLSU), nuclear ribosomal small subunit (nrSSU), partial translation elongation factor1-alpha gene (*tef1*-*α*), RNA polymerase II 2^nd^ largest subunit (*RPB2*), and partial beta-tubulin gene (*tub2*) were amplified using the selected primer pairs: ITS1/ITS4 (White et al. 1990), LR0R/LR5 (Vilgalys and Hester 1990), NS1/NS4 (Hibbett 1996), EF1-728F/EF-2, fRPB2-5f/fRPB2-7cR (Liu et al. 1999), Bt2a/Bt2b (Glass and Donaldson 1995), respectively. The PCR cycling was conducted following the protocol of Song et al. (2023), Barreto et al. (2025), and Zhou et al. (2025). All newly generated sequences in this study were deposited in GenBank and were listed in bold in Table 1.

**Table 1.** Cultures, specimens and NCBI accession numbers included in this study. ^T^ indicates ex-type strains.

**Species**	**Isolate/Culture collection**	**GenBank accession numbers**						**References**
		**ITS**	**LSU**	**tub2**	**tef1**	**nrSSU**	**RPB2**	
*Bellamyces quercus*	CBS 46217^T^	MK810901	MK810788	–	–	–	MK887796	Shen et al. (2020)
*Echinocatena arthrinioides*	CBS 144202	MH107890	MH107937	–	–	–	–	Crous et al. (2018)
*Fuscohilum rhodense*	CBS 121641^T^	MK810909	MK810796	–	–	–	MK887802	Shen et al. (2020)
*Fuscohilum sicilianum*	CBS 105.85^T^	MK810910	MK810797	–	–	–	MN091924	Shen et al. (2020)
*Guizhoumyces acicularis*	GUCC 18195^T^	MZ503724	MZ503757	–	–	MZ503650	MZ546866	Wei et al. (2022)
*Guizhoumyces acicularis*	GUCC 18152	MZ503723	MZ503756	–	–	MZ503649	MZ546865	Wei et al. (2022)
*Helicopsis olivacea*	CBS 728.83	MH861681	MH873393	–	–	AY856925	–	Vu et al. (2019)
*Matsushimaea fasciculata*	CBS 167.97^T^	LT962397	LT962402	–	–	–	–	Unpublished
*Matsushimaea fasciculata*	GUCC 18239	MZ503725	MZ503758	–	–	MZ503651	MZ546867	Wei et al. (2022)
*Matsushimaea monilioides*	CBS 143867^T^	LT883468	LT883469	–	–	–	–	Crous et al. (2018)
*Melnikomyces longisporus*	HUGP 18226^T^	MT731290	MT731291	–	–	MT731289	–	
*Melnikomyces thailandicus*	CBS 145767^T^	MN794374	MN794351	–	–	–	–	Hernández-Restrepo et al. (2020)
*Melnikomyces vietnamensis*	CBS 136209^T^	KJ869156	KJ869213	–	–	–	–	Crous et al. (2014)
*Mycosisymbrium cirrhosum*	GUFCC 18012	KR259883	KR259884	–	–	KR259885	KR349124	Pratibha and Prabhugaonkar (2016)
*Mycosisymbrium cirrhosum*	GUCC 1837	MZ503722	MZ503755	–	–	MZ503648	MZ546864	Wei et al. (2022)
*Neocoleroa cameroonensis*	CBS 129041^T^	MK810902	MK810789	–	–	–	MK887797	Shen et al. (2020)
*Neocoleroa metrosideri*	ICMP 21139^T^	KU131678	KU131677	–	–	–	–	Johnston and Park (2016)
*Neofusicladium eucalypti*	CBS 128216^T^	MK810903	MK810790	–	–	–	MK887798	Shen et al. (2020)
*Neofusicladium eucalypticola*	CBS 141301^T^	MK810904	MK810791	–	–	–	MK887799	Shen et al. (2020)
*Neofusicladium eucalypticola*	CBS 143427	MK810905	MK810792	–	–	–	–	Shen et al. (2020)
*Neofusicladium regnans*	CBS 143411^T^	MG386066	MG386119	–	–	–	–	Shen et al. (2020)
*Neofusicladium regnans*	CBS 144605	MK442628	MK442563	–	–	–	–	Crous et al. (2019)
*Parafusicladium amoenum*	CBS 254.95^T^	MK810906	MK810793	–	–	–	–	Shen et al. (2020)
*Parafusicladium intermedium*	CBS 110746^T^	MK810907	MK810794	–	–	–	MK887800	Shen et al. (2020)
*Parafusicladium paraamoenum*	CBS 141322^T^	MK810908	MK810795	–	–	–	MK887801	Shen et al. (2020)
*Pinaceicola cordae*	CBS 126959^T^	MK810911	MK810798	–	–	–	–	Shen et al. (2020)
*Pinaceicola cordae*	CBS 675.82	MK810912	MK810799	–	–	–	–	Shen et al. (2020)
*Pinaceicola cordae*	CBS 143494	MK810913	MK810800	–	–	–	–	Shen et al. (2020)
*Pinaceicola pini*	CBS 463.82^T^	MK810915	MK810802	–	–	–	MK887804	Shen et al. (2020)
*Pinaceicola pini*	CBS 462.82	MK810914	MK810801	–	–	–	MK887803	Shen et al. (2020)
*Pseudosigmoidea excentrica*	CBS 469.95^T^	HQ667543	KF282669	–	–	NG_065605	–	Samerpitak et al. (2014)
*Pseudosigmoidea ibarakiensis*	NBRC 107891^T^	LC146758	LC146759	–	–	NG_078726	–	Unpublished
*Scolecobasidium acanthi*	LC19368^T^	OQ448957	OQ448949	OQ442218	OQ442215	–	–	Song et al. (2023)
*Scolecobasidium aegiceratis*	LC19369^T^	OQ448958	OQ448950	OQ442219	OQ442216	–	–	Song et al. (2023)
*Scolecobasidium aegiceratis*	LC19370	OQ448959	OQ448951	OQ442220	OQ442217	–	–	Song et al. (2023)
*Scolecobasidium ailanthi*	MFLU 18-2110	MK347731	–	MK412881	–	–	–	Jayasiri et al. (2019)
*Scolecobasidium ailanthi*	MFLUCC 17-0923^T^	MK347730	MK347947	MK412883	–	–	–	Jayasiri et al. (2019)
*Scolecobasidium anellii*	CBS 284.64^T^	FR832477	KF156138	KF156184	KF155995	–	–	Martin-Sanchez et al. (2012)
*Scolecobasidium anellii*	CBS 284.64^T^	FR832477	KF156138	–	–	NG_062993	KF282684	Martin-Sanchez et al. (2012)
*Scolecobasidium anomalum*	CBS 131816^T^	HE575201	KF156137	KF156194	KF155986	–	–	Samerpitak et al. (2015)
*Scolecobasidium anomalum*	CBS 131816^T^	HE575201	KF156137	–	–	NG_062986	HE575205	Samerpitak et al. (2015)
*Scolecobasidium aquaticum*	CBS 140316^T^	KX668258	KX668259	–	–	–	–	Samerpitak et al. (2017)
*Scolecobasidium bacilliforme*	CBS 100442^T^	KP798632	KP798635	KT272059	KT272070	–	–	Samerpitak et al. (2015)
*Scolecobasidium blechni*	CBS 146055^T^	MN562134	MN567641	MN556843	MN556826	–	–	Martin-Sanchez et al. (2012)
*Scolecobasidium blechni*	CBS 146055^T^	MN562134	MN567641	–	–	–	–	Martin-Sanchez et al. (2012)
*Scolecobasidium camellicola*	GUCC 18242^T^	MZ503728	MZ503761	MZ546907	MZ546874	–	–	Wei et al. (2022)
*Scolecobasidium capsici*	CBS 142096^T^	KY173427	KY173518	–	–	–	–	Crous et al. (2016)
*Scolecobasidium coiledmyces*	GUCC 18245^T^	MZ503731	MZ503764		MZ546877	MZ503657	MZ546910	Wei et al. (2022)
*Scolecobasidium constrictum*	CBS 202.27^T^	MH854929	MH866423	KF156161	KF156003	–	–	Machouart et al. (2014)
*Scolecobasidium constrictum*	CBS 211.53^T^	HQ667519	KF156148	–	–	NG_062994	KF282686	Machouart et al. (2014)
*Scolecobasidium cordanae*	CBS 412.51	HQ667540	KF156123	KF156200	KF155980	–	–	Vu et al. (2019)
*Scolecobasidium cordanae*	CBS 475.80^T^	KF156022	KF156122	KF156197	KF155981	–	–	Machouart et al. (2014)
*Scolecobasidium crassihumicola*	CBS 120700	KJ867429	KJ867430	KJ867433	KJ867428	–	–	Samerpitak et al. (2015)
*Scolecobasidium crassihumicola*	CBS 120700	KJ867429	KJ867430	–	–	–	–	Samerpitak et al. (2015)
*Scolecobasidium dracaenae*	CBS 141323^T^	KX228283	KX228334	–	KX228377	–	–	Vu et al. (2019)
*Scolecobasidium echinulatum*	GUCC 18247^T^	MZ503733	MZ503766	MZ546912	MZ546879	–	–	Wei et al. (2022)
*Scolecobasidium echinulatum*	GUCC 18248	MZ503734	MZ503767	MZ546913	MZ546880	–	–	Wei et al. (2022)
*Scolecobasidium ellipsoideum*	CBS 131796^T^	MN077367	–	–	–	–	–	Shen et al. (2020)
*Scolecobasidium ellipsoideum*	GUCC 18264	MZ503750	MZ503783	MZ546929	MZ546896	–	–	Shen et al. (2020)
*Scolecobasidium endophyticum*	P6447^T^	MN365755	MN308484	–	PX047958	–	–	Verma (2014)
*Scolecobasidium endophyticum*	P6546	MN310251	MN308559	–	PX047960	–	–	Verma (2014)
*Scolecobasidium ferulica*	IRAN3232C^T^	MF186874	MH400207	–	–	–	–	Safaie et al. (2024)
*Scolecobasidium gamsii*	CBS 239.78^T^	KF156019	KF156150	KF156190	KF155982	–	–	Samerpitak et al. (2015)
*Scolecobasidium gamsii*	CBS 239.78^T^	KF156019	KF156150	–	–	NG_062991	–	Samerpitak et al. (2015)
*Scolecobasidium globale*	CBS 119644^T^	KF961086	KF961097	KF961065	KF961075	–	–	Samerpitak et al. (2015)
*Scolecobasidium globale*	CBS 135924	KF961092	KF961104	KF961070	KF961079	–	–	Samerpitak et al. (2015)
*Scolecobasidium guangxiensis*	SS23^T^	MK934570	MK956169	–	–	–	–	Verma (2014)
*Scolecobasidium guangxiensis*	X22	MK961215	MK961247	–	–	–	–	Crous et al. (2019)
*Scolecobasidium guizhouense*	ZY22.022 ^T^	OR680501	OR680568	OR843206	–	–	–	Verma (2014)
*Scolecobasidium guizhouense*	ZY22.023	OR680502	OR680569	OR843207	–	–	–	Verma (2014)
*Scolecobasidium guizhouense*	ZY22.024	OR680503	OR680570	OR843208	–	–	–	Verma (2014)
*Scolecobasidium helicteris*	NFCCI 4310^T^	MK014833	–	MK321318	–	–	–	Verma (2014)
*Scolecobasidium humicola*	CBS 116655^T^	HQ667521	KF156124	KF156195	KF155984	–	–	Samerpitak et al. (2014)
*Scolecobasidium icarus*	CBS 116645	HQ667525	–	LM644604	–	–	–	Samerpitak et al. (2014)
*Scolecobasidium icarus*	CBS 536.69^T^	HQ667524	KF156132	KF156174	KF156009	–	–	Samerpitak et al. (2015)
*Scolecobasidium icarus*	CBS 536.69^T^	HQ667524	KF156132	–	–	NG_062989	KF282700	Samerpitak et al. (2015)
*Scolecobasidium inverellipsoidisporum*	ZY22.027 T	OR680506	OR680573	–	OR858905	–	–	Zhang et al. (2024a)
*Scolecobasidium inverellipsoidisporum*	ZY22.029	OR680508	OR680575	–	–	–	–	Zhang et al. (2024a)
*Scolecobasidium laurentii*	NFCCI 5982	PV029878	PV029879	PV023970	PV023971	–	–	Zalar et al. (2022)
*Scolecobasidium lascauxense*	CBS 131815^T^	FR832474	KF156136	KF156183	KF155994	–	–	Schoch et al. (2014)
*Scolecobasidium lascauxense*	CBS 423.64	HQ667523	KF156131	KF156173	KF156008	–	–	Schoch et al. (2014)
*Scolecobasidium leishanicola*	GUCC 18259	MZ503745	MZ503778	MZ546924	MZ546891	–	–	Wei et al. (2022)
*Scolecobasidium leishanicola*	HGUP1808^T^	MK377301	MK377073	–	–	–	–	Suthar et al. (2025)
*Scolecobasidium longiphorum*	CBS 435.76^T^	KF156038	KF156135	KF156182	KF155978	–	–	Samerpitak et al. (2014)
*Scolecobasidium macrozamiae*	CBS 102491	KF156021	KF156152	–	KF155983	–	–	Samerpitak et al. (2014)
*Scolecobasidium macrozamiae*	CBS 137971^T^	KJ869123	KJ869180	–	–	–	–	Crous et al. (2014)
*Scolecobasidium minimum*	CBS 510.71^T^	HQ667522	KF156134	KF156172	KF156007	–	–	Samerpitak et al. (2014)
*Scolecobasidium minimum*	CBS 119792	KF156027	KF156133	–	KT272073	–	–	Samerpitak et al. (2014)
*Scolecobasidium mirabilis*	CBS 413.51^T^	HQ667536	KF156140	KF156164	KF156001	–	–	Samerpitak et al. (2014)
*Scolecobasidium musae*	CBS 729.95^T^	KF156029	KF156144	KF156171	KF155999	–	–	Samerpitak et al. (2014)
*Scolecobasidium musicola*	CPC 37308	MW063428	–	MW071116	MW071096	–	–	Crous et al. (2021)
*Scolecobasidium musicola*	CPC 37309	MW063429	–	MW071117	MW071097	–	–	Crous et al. (2021)
*Scolecobasidium musicola*	CPC 32927	NR_160360	NG_064553	MH327898	MH327887	–	–	Crous et al. (2018)
*Scolecobasidium obovoideum*	GUCC 18246^T^	MZ503732	MZ503765	MZ546911	MZ546878	–	–	Wei et al. (2022)
*Scolecobasidium olivaceum*	CBS 137170^T^	LM644521	LM644564	LM644605	KT272067	–	–	Giraldo et al. (2014)
*Scolecobasidium pandanicola*	CBS 140660^T^	KT950850	KT950864	–	–	–	–	Verma (2014)
*Scolecobasidium parahumicola*	ZY22.025 T	OR680504	OR680571	OR843209	OR858903	–	–	Zhang et al. (2024a)
*Scolecobasidium parahumicola*	ZY22.026	OR680505	OR680572	OR843210	OR858904	–	–	Zhang et al. (2024a)
*Scolecobasidium phaeophorum*	CBS 206.96^T^	KP798631	KP798634	KT272062	KT272098	–	–	Samerpitak et al. (2015)
*Scolecobasidium podocarpi*	CBS 143174^T^	MG386032	MG386085	–	–	–	–	Verma (2014)
*Scolecobasidium podocarpicola*	CBS 146057^T^	MN562138	MN567645	–	–	–	–	Crous et al. (2019)
*Scolecobasidium ramosum*	CBS 137173^T^	LM644524	LM644567	MZ546928	KT272069	–	–	Giraldo et al. (2014)
*Scolecobasidium ramosum*	CBS 137171	LM644522	LM644565	LM644606	KT272068	–	–	Samerpitak et al. (2016)
*Scolecobasidium robustum*	CBS 112.97^T^	KP798633	KP798636	KT272060	KT272071	–	–	Samerpitak et al. (2015)
*Scolecobasidium sexuale*	CBS 131965	KF156017	KF156119	KF156188	KF155977	–	–	Samerpitak et al. (2014)
*Scolecobasidium sexuale*	CBS 135765^T^	KF156018	KF156118	KF156189	KF155976	–	–	Samerpitak et al. (2014)
*Scolecobasidium spiralihyphum*	GUCC 18245^T^	MZ503731	MZ503764	MZ546910	MZ546877	–	–	Wei et al. (2022)
*Scolecobasidium terrestre*	CBS 211.53^T^	NR145365	NG058014	KF156187	KF156005	–	–	Machouart et al. (2014)
*Scolecobasidium terreum*	CBS 203.27^T^	HQ667544	–	HQ877665	–	–	–	Samerpitak et al. (2014)
*Scolecobasidium tshawytschae*	CBS 100438^T^	HQ667562	KF156126	KF156180	KF155990	–	–	Samerpitak et al. (2014)
*Scolecobasidium tshawytschae*	CBS 228.66	KF156016	KF156128	KF156179	KF155992	–	–	Samerpitak et al. (2014)
*Scolecobasidium variabile*	NBRC 32268	DQ307334	EU107310	–	DQ307356	–	–	Dela Cruz et al. (2006)
*Scolecobasidium verrucaria*	GUCC 18240^T^	MZ503726	MZ503759	MZ546905	MZ546872	–	–	Wei et al. (2022)
*Scolecobasidium verrucosum*	CBS 383.81^T^	KF156015	KF156129	KF156185	KT272099	–	–	Samerpitak et al. (2014)
***Scolecobasidium yunnanense***	**WF6-2b**	**PX984820**	**PX984826**	–	**PZ129585**	–		**In this study**
***Scolecobasidium yunnanense***	**WF6-2a^T^**	**PX984821**	**PX984827**	–	**PZ129586**	–	–	**In this study**
*Scolecobasidium zunyiense*	GUCC 18241^T^	MZ503727	MZ503760	MZ546906	MZ546873	–		Wei et al. (2022)
*Sterila eucalypti*	CBS 144019^T^	MK810918	MK810805	–	–	–	MK887807	Shen et al. (2020)
*Sterila eucalypti*	CPC 14942	MK810916	MK810803	–	–	–	MK887805	Shen et al. (2020)
*Sterila eucalypti*	CPC 14943	MK810917	MK810804	–	–	–	MK887806	Shen et al. (2020)
*Sympoventuria capensis*	CBS 120136^T^	MK810921	MK810808	–	–	NG_061163	MK887810	Shen et al. (2020)
*Sympoventuria capensis*	CPC 12839	MK810922	MK810809	–	–	–	MK887811	Shen et al. (2020)
*Sympoventuria capensis*	CPC 12840	MK810923	MK810810	–	–	–	MK887812	Shen et al. (2020)
*Sympoventuria melaleucae*	CBS 143407^T^	MG386059	MG386112	–	–	–	–	Unpublished
*Troposporella fumosa*	CBS 351.94	MK810924	MH874121	–	–	–	–	Shen et al. (2020)
*Troposporella monilipes*	MUCL 19867	DQ351723	AY856871	–	–	AY856920	–	Tsui and Berbee (2010)
*Tyrannosorus lichenicola*	CBS 144018^T^	MK810953	MK810838	–	–	–	MK887840	Shen et al. (2020)
*Venturia saliciperda*	CBS 480.61^T^	MK811007	MK810891	–	–	–	MK887886	Shen et al. (2020)
*Veronaeopsis simplex*	CBS 588.66^T^	EU041820	EU041877	–	–	NG_061164	MN091925	Arzanlou et al. (2007)
*Verruconis calidifluminalis*	CBS 125818^T^	AB385698	KF156108	KF156202	KF155959	–	–	Yarita et al. (2010)
*Verruconis gallopava*	CBS 437.64^T^	HQ667553	KF156112	–	–	NG_062995	KF282689	Machouart et al. (2014)
*Verruconis mangrovei*	NFCCI-4390T	MN782361	NG_073757	–	–	NG_070311	MN231836	Unpublished
*Verruconis pseudotricladiata*	YMF1.04915^T^	MK244396	MK248270	–	–	–	–	Qiao et al. (2019)
*Verruconis panacis*	CBS 142802^T^	MF536882	MF536880	–	–	–	–	
***Yunnanomyces didymosporus***	**KO1-6a1**	**PX984822**	**PX984828**	–	–	**PX984824**	**PZ129583**	**In this study**
***Yunnanomyces didymosporus***	**KO1-2^T^**	**PX984823**	**PX984829**	–	–	**PX984825**	**PZ129584**	**In this study**
*Yunnanomyces hagahagaensis*	CPC 47671^T^	PQ498953	PQ499002	–	–	–	PQ497730	Unpublished
*Yunnanomyces mangrovei*	SNT62^T^	–	PP621124	–	–	PP639251	PP780209	Zhang et al. (2024a)
*Yunnanomyces muriformis*	GZAAS 24-0053^T^	PP657299	PP657341	–	–	–	–	Liu et al. (2024)
*Yunnanomyces narathiwatensis*	MFLUCC 24-0574	PV271878	PV271920	–	–	–	PV340525	Karimi et al. (2025)
*Yunnanomyces panamaensis*	CPC 46559	PQ498939	PQ498988	–	–	–	PQ497727	Crous et al. (2024)
*Yunnanomyces pandanicola*	MFLUCC 17-2260^T^	MH388369	MH376743	–	–	MH388333	MH412736	Tibpromma et al. (2018)
*Yunnanomyces phoenicis*	MFLUCC 19-0254^T^	–	MK976738	–	–	MK976740	MK986484	Zhang et al. (2019)
*Yunnanomyces phoenicis*	MFLUCC 19-0253	–	MK976737	–	–	MK976739	MK986483	Zhang et al. (2019)
*Yunnanomyces sexualis*	ZY22.033^T^	OR680512	OR680579	–	–	–	OR842929	Zhang et al. (2024b)
*Yunnanomyces sexualis*	ZY22.034	OR680513	OR680580	–	–	–	OR842930	Zhang et al. (2024b)
*Yunnanomyces syagri*	CCMB 739^T^	PP718673	PP718674	–	–	PP718675	PP747050	Barreto et al. (2025)

### Phylogenetic analyses

New sequences, deposited in GenBank (http://www.ncbi.nlm.nih.gov/genbank/) (Table 1), were aligned with additional sequences retrieved from GenBank (Table 1) using BioEdit 7.0.5.3 (Hall 1999) and MAFFT v.74 (http://mafft.cbrc.jp/alignment/server/, Katoh et al. 2017). Sequences of *Scolecobasidium* were adopted mainly from nLSU + ITS + *tub2* + *tef1*-*α* tree topologies established by Song et al. (2023). Sequences of *Yunnanomyces* were adopted mainly from ITS + nLSU + nrSSU + *RPB2* tree topologies established by Barreto et al. (2025). Maximum Likelihood (ML) and Bayesian Inference (BI) methods were used for phylogenetic analyses. The ML analysis was carried out with RAxML version 8.2.12 (Stamatakis 2014), which statistical support values were obtained using rapid bootstrapping with 1,000 replicates, with default settings for other parameters and the best-fit models. The BI tree reconstruction was carried out with MrBayes v. 3.2.5 (Ronquist and Huelsenbeck 2003), with the best-fit partitioning scheme and substitution model determined by using ModelFinder (Kalyaanamoorthy et al. 2017; Zhang et al. 2020) via the “greedy” algorithm, and branch lengths were estimated as “linked” and AICc. Four Markov chains were run for two runs from random starting trees for 10 million generations and trees were sampled every 1,000 generations. The burn-in was set to discard 25% of the trees. A majority rule consensus tree of all remaining trees was calculated. Branches that received bootstrap support for Maximum Likelihood (ML) and Bayesian Posterior Probabilities (BPP) greater than or equal to 75% (ML) and 0.95 (BPP) were considered as significantly supported.

## Results

### Phylogeny

**Figure 1.**
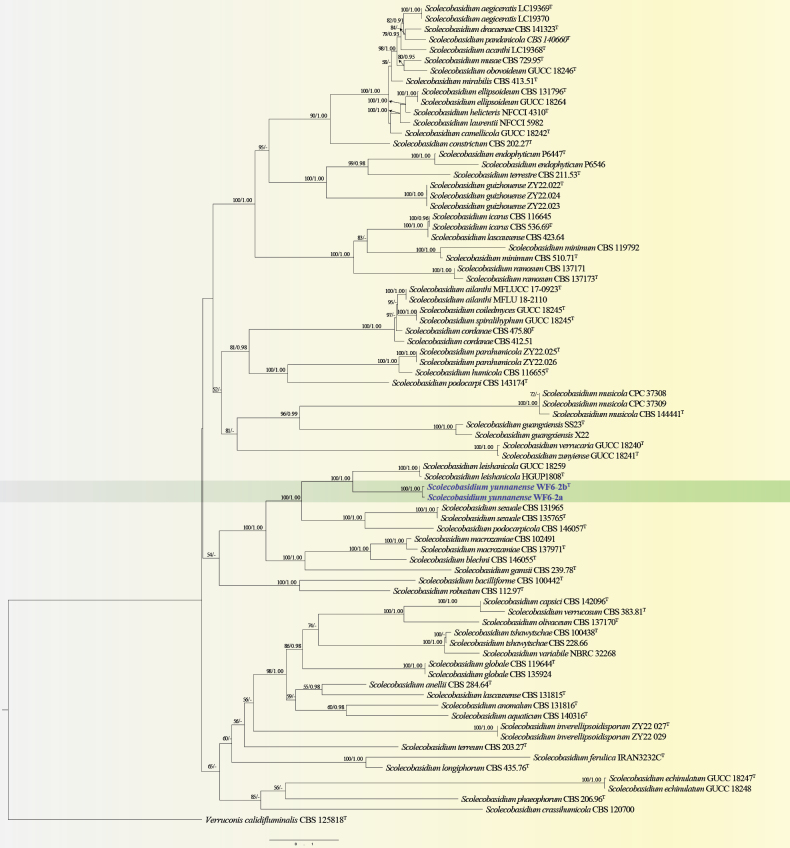
Maximum Likelihood (ML) tree illustrating the phylogeny of the genus *Scolecobasidium*, based on a combined ITS + nLSU + *tub2* + *tef1*-*α* dataset. Branches are labelled with parsimony bootstrap values (ML) higher than 75% and Bayesian Posterior Probabilities (BPPs) more than 0.95. ^T^ indicates ex-type strains.

The *Scolecobasidium* dataset comprised four loci (nLSU + ITS + *tub2* + *tef1*-*α*) from 78 strains representing 57 species. Sequences of *Verruconis calidifluminalis* (Yarita et al.) Samerp. & de Hoog was used as the outgroup, as in Song et al. (2023). The dataset had an aligned length of 2,990 characters, including 830 bp of ITS, 890 of nLSU, 588 of *tub2*, and 682 of *tef1*-*α*. ModelFinder suggested models were GTR + F + I + G4 for ITS, nLSU, *tef1*-*α*, and GTR + F + G4 for *tub2*, for the Bayesian analysis. The BI analysis resulted in a concordant topology with an average standard deviation of split frequencies of 0.006261. In our phylogenetic tree (Fig. 1), two strains (WF6-2b and WF6-2a) representing a new species, *Scolecobasidium yunnanense*, were robustly placed within the *Scolecobasidium*, forming a distinct, well-supported monophyletic lineage (100% ML/1.00 BPP), clearly separated from all other known species. Nearly all accepted species of *Scolecobasidium* form distinct, supported lineages, consistent with the phylogenetic topology proposed by Song et al. (2023). Furthermore, *Scolecobasidium yunnanense* is closely related to *S. leishanicola* T.P. Wei & Y.L. Jiang, together form a strongly supported clade (100% ML/1.00 BPP).

The *Yunnanomyces* dataset comprised four loci (ITS + nLSU + nrSSU + *RPB2*) from 65 strains representing 51 species. Two species of *Tyrannosorus lichenicola* Crous, M. Shen & Y. Zhang ter and *Venturia saliciperda* Nüesch were used as the outgroups. The dataset had an aligned length of 5,164 characters, including 808 sequences of ITS, 881 of nLSU, 1,111 of nrSSU, and 2,364 of *RPB2*. ModelFinder suggested models were GTR + F + I + G4 for ITS, nLSU, RPB2, and SYM+I for nrSSU, for the Bayesian analysis. The BI analysis resulted in a concordant topology with an average standard deviation of split frequencies of 0.006261. In the combined (ITS + LSU + nrSSU + RPB2) dataset, our strains (KO1-6a1 and KO1-2) representing a new species, *Yunnanomyces didymosporus*, form a fully supported (100% ML/1.00 BPP) unique lineage sister to *Y. hagahaensis* Crous. And all accepted species of *Yunnanomyces* form distinct, supported lineages with in *Sympoventuriaceae*.

In addition, BLAST analyses of respective gene fragments for *Scolecobasidium yunnanense* and *Yunnanomyces didymosporus* were carried out via the NCBI nucleotide database, with all results summarized in Table 2.

**Table 2.** BLAST analyses of respective gene fragments for the holotype of *Scolecobasidium yunnanense* and *Yunnanomyces didymosporus*.

**Species**	**Marker**	**BLAST analyses**			
		**Accession No**.	**Query Cover (%)**	**Percent Ident (%)**	**Reference**
*Scolecobasidium yunnanense* (CFCC73245)	ITS	OQ076495	96	97.42	Papan et al. 2023
	nLSU	MZ503778	97	97.99	Wei et al. 2022
	*tef1*-*α*	KF155976	95	85.14	Samerpitak et al. 2014
*Yunnanomyces didymosporus* (CFCC73246)	ITS	PP718673	96	94.48	Barreto et al. 2025
	nLSU	PV271920	97	97.17	Karimi et al. 2025
	nrSSU	NG245372	92	98.54	Zhang et al. 2024a
	*RPB2*	PP747050	96	88.98	Barreto et al. 2025

### Taxonomy

#### Scolecobasidium yunnanense

Taxon classificationFungiVenturialesSympoventuriaceae

L.H. Zhu, Q.Y. Zhang & D.W. Li sp. nov.

62BB1ECD-C27C-565D-8582-8E1B853DBB1E

862361

##### Etymology.

Yunnanense (Lat.) indicates that this species was first collected from Yunnan Province, China.

##### Type.

China • Yunnan Province, Xishuangbanna Prefecture, Mengla County, Menglun Town, Xishuangbanna Tropical Botanical Garden (21°55'0.12"N, 101°15'E), isolated from the dead leaves of the *Washingtonia filifera* (T.Moore & Mast.) H.Wendl. ex de Bary, Nov. 2024, L.H. Zhu & D.W. Li, holotype, CFCC73245(=WF6-2b). The holotype is a living strain being maintained via lyophilization at the China Forestry Culture Collection Center (CFCC), Chinese Academy of Forestry, Beijing, China.

##### Description.

Asexual morph on PDA: Mycelium consisting of branched, septate, hyaline to pale brown, smooth, and thick-walled hyphae. Conidiophores solitary, erect, brown, smooth, arising from superficial hyphae, subcylindrical, straight to geniculous, septate, unbranched (10–)17–42.5 × 1.5–3 µm, often reduced to conidiogenous cells, bearing a few conidia near the apex. Conidiogenous cells brown, smooth, 2.5–6.8 × 1–2 µm, terminal and lateral on conidiophores, containing several apical, apiculate denticles. **Conidia** produced directly from the sides or apices of hyphae, smooth-walled, subhyaline to mid-brown, ellipsoidal, oblong or fusiform, tapering towards the base, mostly 2-septate, rarely 1 or 3-septate, sometimes slightly constricted at the septa, (8.0–)8.3–12.6(–12.8) × (1.8–)2.0–3.5(–3.7) μm (av. ± SD = 10.5 ± 0.8 × 2.6 ± 0.2 μm, n = 60).

##### Culture characteristics.

Colonies on PDA 2.48–2.6 cm diam after 15 days at 25 °C, smooth to felty, dry, surface grayish brown to dark brown, reverse saddle brown. Conidia can be observed on the culture surface as early as the 5^th^ day post-inoculation.

##### Additional material examined.

China • Yunnan Province, Xishuangbanna Prefecture, Mengla County, Menglun Town, Xishuangbanna Tropical Botanical Garden (21°55'0.12"N, 101°15'E), isolated from the dead leaves of the *Washingtonia filifera*, Nov. 2024, L.H. Zhu & D.W. Li. WF6-2a, which is stored at the Forest Pathology Laboratory, Nanjing Forestry University.

##### Notes.

In this study, two strains isolated from palm represent a novel species of *Scolecobasidium*, distinguished from previously described species by both morphological and phylogenetic evidence. Phylogenetically, the new species in this study, *Scolecobasidium yunnanense*, related to *S. leishanicola* (HGUP1808), and formed a fully supported clade. *Scolecobasidium leishanicola* (as *Ochroconis terricola*) was previously recorded from soil in China (Zhang et al. 2020). And *S. leishanicola* can be easily distinguished to *S. yunnanense* by its subhyaline to pale brown mycelia obvious conidiophores and 1-septate conidia (Wei et al. 2022). Morphologically, *Scolecobasidium yunnanense* differs from most species in the genus by producing 2–3-septate conidia, whereas the majority of *Scolecobasidiums* species typically possess 1-septate conidia. Although *S. tshawytschae* (Doty and D.W. Slater) McGinnis & Ajello (CBS 100438) are similar in having 2–3-septate conidia, it can be distinguished from *S. yunnanense* by its larger conidia, 12.5–22 × 5–6 µm vs. 8.3–12.6 × 2.0–3.5 μm and the presence of acrogenous chlamydospores (Wei et al. 2022).

#### Yunnanomyces didymosporus

Taxon classificationFungiVenturialesSympoventuriaceae

L.H. Zhu, Q.Y. Zhang & D.W. Li sp. nov.

86FB8E82-39E1-527B-A831-F6D909B22735

862362

##### Etymology.

Didymosporus (Lat.) referring to the predominantly 2-celled conidia.

##### Type.

China • Yunnan Province, Xishuangbanna Prefecture, Mengla County, Menglun Town, Xishuangbanna Tropical Botanical Garden (21°55'0.12"N, 101°15'E), isolated from dead leaves of the *Kentiopsis oliviformis* (Brongn. & Gris) Hodel & C.F.Barrett, Nov. 2024, L.H. Zhu & D.W. Li., holotype, CFCC73246 (=Ko1-2). Holotype is a living strain being maintained via lyophilization at the China Forestry Culture Collection Center (CFCC), Chinese Academy of Forestry, Beijing, China.

##### Description.

Asexual morph on PDA: ***Mycelium*** flat, immersed when mature, composed of septate, branched, subhyaline to pale brown hyphae, 1–3 μm wide, with the aged mycelium monilioid. ***Conidiophores*** arising from the aerial hyphae or hyphal ropes, aseptate or septate, usually reduced to conidiogenous cells, 0(–1)-septate. ***Conidiogenous cells*** are integrated, usually intercalary, hyaline, thin-walled, smooth, straight to sinuous, 4.74–11.2 × 1.82–6.12 μm, producing conidia in acropetal chains through blastic conidiogenesis; ***Conidia*** arthric, ellipsoidal to clavate, smooth, some with slightly truncate ends, hyaline to pale brown, mostly 1-septate, rarely 0 or 2-septate, arranged in chains containing up to 8 conidia, secession schizolytic, leaving a dark brown to black hilum on one or both ends, 1–2-septate conidia mostly constricted at the septation, (5.0–)6.1–11.9(–12.1) × (1.7–)2.0–4.2(–4.6) μm (mean ± SD = 7.9 ± 0.3 × 2.7 ± 0.2 μm, n = 60). Chlamydospores and sexual morph not observed.

##### Cultural characters.

Colonies on PDA 3.38–3.52 cm diam after 30 days at 25 °C, circular, flat, velvety, with brown filamentous margin, obverse gray-green to brown, reverse dark brown. Conidia can be observed on the culture surface as early as the 5^th^ day post-inoculation.

##### Additional material examined.

China • Yunnan Province, Xishuangbanna Prefecture, Mengla County, Menglun Town, Xishuangbanna Tropical Botanical Garden (21°55'0.12"N, 101°15'E), isolated from dead leaves of the *Kentiopsis oliviformis*, Nov. 2024, L.H. Zhu & D.W. Li., Ko1-6a1, which is stored at the Forest Pathology Laboratory, Nanjing Forestry University.

##### Notes.

*Yunnanomyces didymosporus* is recognized as a new species based on phylogenetic distinctiveness and morphological differences. Phylogenetically, *Yunnanomyces didymosporus* formed an independent lineage in the *Yunnanomyces* clade and grouped with *Y. hagahagaensis* with strong support. While based on the ITS sequence data, *Yunnanomyces didymosporus* and *Y. hagahagaensis* (CPC 47671) showed a difference of 17 base pairs, with a divergence rate of over 2%. Morphologically, *Y. hagahagaensis* can be easily distinguished from *Y. didymosporus* by its larger conidia, 20–27 × 2.5–3 μm vs. 6.1–11.9 × 2.0–4.2 μm (Crous et al. 2024). Furthermore, *Yunnanomyces didymosporus* exhibits obvious morphological discrepancies from all other accepted species within *Yunnanomyces*. A comparative synopsis summarizing the asexual morphological features of all known *Yunnanomyces* taxa is presented in Table 2.

## Discussion

Despite existing investigations into fungal diversity on palms, novel diseases and fungal species are still discovered and reported (Peng et al. 2015; Zhou et al. 2025). In this study, two novel fungal species belonging to *Scolecobasidium* and *Yunnanomyces* were isolated from palm hosts based on morphological and phylogenetic evidence.

The genus *Scolecobasidium* exhibits high interspecific ITS sequence variability (Hao et al. 2013). And most congeneric species have publicly available ITS reference sequences, providing robust molecular evidence for species delimitation and identification within this genus. The newly described species *S. yunnanense* forms a well-supported, distinct phylogenetic clade in the phylogenetic tree. Furthermore, Scolecobasidium species show wide geographical distribution and can colonize a broad spectrum of substrates. Members of this genus have been recorded as pathogens of plants, animals, and humans (Barron and Busch 1962; Hamayun et al. 2009). Additionally, some species have also been isolated from marine environments, petroleum products, soil and painted surfaces (Barron and Busch 1962; Roy et al. 1962; Punithalingam and Spooner 2011; Hao et al. 2013). In the present study, *S. yunnanense* was isolated from palm trees, which further indicates that *Scolecobasidium* species are adaptable to an epiphytic lifestyle.

*Yunnanomyces* is a small genus in the family *Sympoventuriaceae*. Previous studies have demonstrated that it is commonly associated with monocotyledonous plants (Crous et al. 2024; Zhang et al. 2024a). The original generic concept of *Yunnanomyces* is that the conidia are muriform (dictyoconidia) (Tibpromma et al. 2018). Among these nine taxa in *Yunnanomyces*, two taxa develop only sexual state (*Y. mangrovei* and *Y. sexualis*) (Crous et al. 2024; Zhang et al. 2024a, 2024b). With regard to the asexual morphology of *Yunnanomyces*, most species produce dark conidia that are subglobose to irregular ellipsoidal and muriform. Three species form subcylindrical conidia in branched chains, viz. 0-1 septate conidia (*Y. hagahagaensis*), didymoconidia (*Y. panamaensis*), 1(-2) septate conidia (*Y. didymosporus*). A synopsis of the asexual morphs of *Yunnanomyces* species is provided in Table 3.

**Table 3.** A synopsis of the asexual morphs of *Yunnanomyces* species.

**Species**	**Conidiophores (µm)**	**Conidiogenous cells (µm)**	**Conidia (µm)**	**References**
***Y. didymosporus*** (CFCC73246)	Arising from the aerial hyphae or hyphal ropes, aseptate or septate, usually reduced to conidiogenous cells, 0(–1)-septate.	Integrated, smooth, straight to sinuous, 4.74–11.2 × 1.82–6.12 μm	Ellipsoidal to clavate, hyaline to pale brown, arranged in chains containing up to 8 conidia, 0–2-septate, 6.1–11.9 × 2.0–4.2 μm	In this study
*Y. hagahaensis* (CBS H-25492)	Micronematous to semi-ma cronematous, subcylindrical, erect, 3–40 × 1.5–3 μm	Polyblastic, sympodial, integrated, terminal, subcylindrical, pale brown, 3–25 × 1.5–2.5 μm	Subcylindrical, branched chains, 0–1-septate, pale brown, guttulate, 20–27 × 2.5–3 μm	Crous et al. (2024)
*Y. muriformis* (MFLU 24-0179)	Micronematous to semi-ma cronematous, mononematous, 1.5–3 μm wide	Monoblastic, integrated, terminal, subhyaline to pale brown	Subglobose to irregular ellipsoidal, muriform, verrucose, golden brown to golden dark brown, 11–19 × 7–11 μm	Liu et al. (2024)
Y. narathiwatensis (MFLU 24-0490)	Semi-macronematous, mostly reduced to conidiogenous cells, pale brown to dark brown, smooth, thick-walled	Integrated, determinate, holoblastic, monoblastic, terminal, cylindrical, brown to dark brown, 3–8 μm	Acrogenous, solitary or arranged in a small chain, subglobose to ellipsoidal or obovoid, muriform, brown to black, thick-walled, comprises 14–40 cells per conidium, apical row containing 1–3 cells, 22–29 × 14–19.8 μm	Karimi et al. (2025)
*Y. panamaensis* (CBS H-25469)	Semi-macronematous, erect, unbranched, subcylindrical, 2–4-septate, 25–60 × 2.5–3 μm	Polyblastic, integrated, terminal, darker brown slightly rough ened, 22–30 × 3 μm	Ellipsoid, 1-septate, brown, finely roughened, 5–6(–7) × 2–2.5 μm	Crous et al. (2024)
*Y. pandanicola* (HKAS 99611)	Macro, semi or micronema tous, fasciculate, unbranched	2.5–5 × 1.5–5 μm	Globose to broadly oval, muriform, yellow-brown, 20–25 × 13–18 μm	Tibpromma et al. (2018)
*Y. phoenicis* (MFLU 19-0811)	Semi-macronematous	0.7–1.2 μm	Globose to broadly ellipsoidal, mu riform, brown, 18–34 × 12–22 μm	Zhang et al. (2019)
Y. syagri (HUEFS 276450)	Macro, semi or microne matous, branched, 15–24 × 2.5–4 μm	5–7.5 × 1.3–2.5 μm	Globose to subglobose, ellipsoidal, muriform, pale brown to brown, 15–33 × 13–17 μm	Barreto et al. (2025)

**Figure 2.**
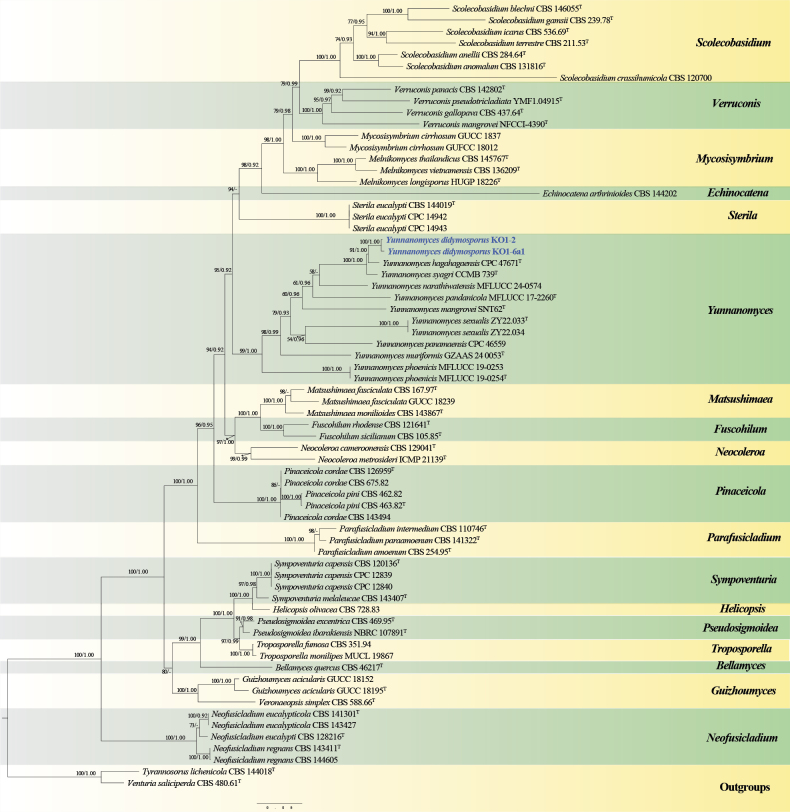
Maximum Likelihood (ML) tree illustrating the phylogeny of the order *Sympoventuriaceae*, based on a combined ITS + nLSU + nrSSU + *RPB2* dataset. Branches are labelled with parsimony bootstrap values (ML) higher than 75% and Bayesian Posterior Probabilities (BPPs) more than 0.95. ^T^ indicates ex-type strains.

**Figure 3.**
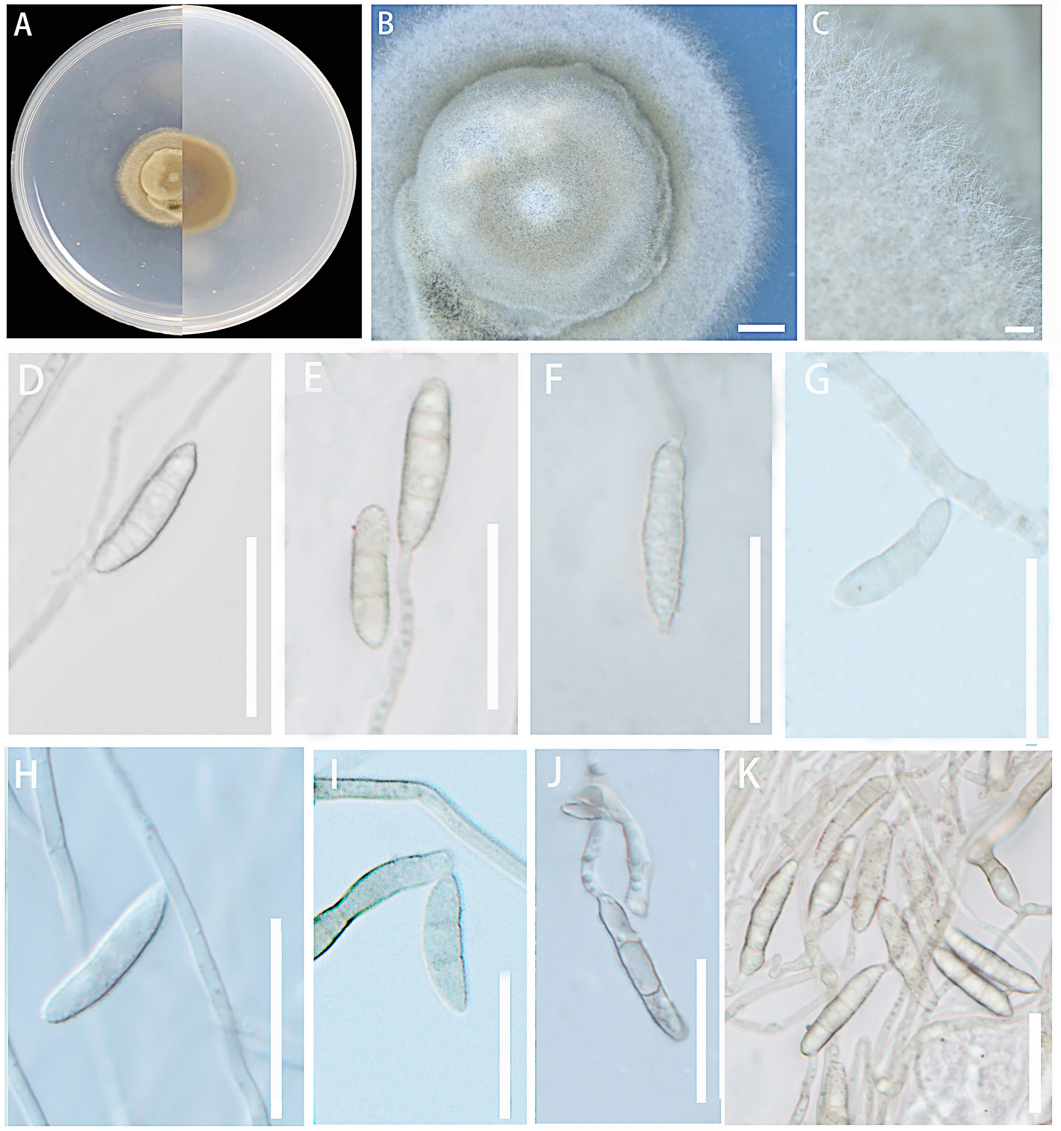
*Scolecobasidium yunnanense* (WF6-2b=73245). **A**. PDA culture colony; **B, C**. Hyphae on the colony, observed under a stereomicroscope; **D–K**. Conidiophores, conidiogenous cells, and conidia. Scale bars: 2 mm (**B**); 0.2 mm (**C**); 10 μm (**D–K**).

**Figure 4.**
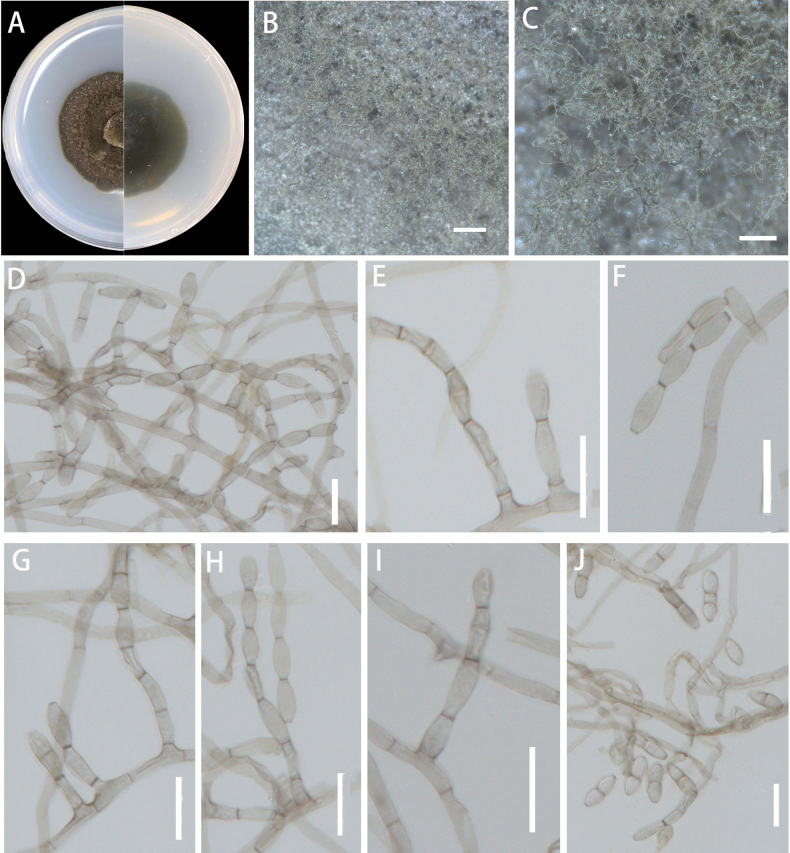
*Yunnanomyces didymosporus* (KO1-2=73246). **A**. PDA culture colony; **B, C**. Hyphae on the colony, observed under a stereomicroscope; **D**. Conidiophores and conidial chains arranged on conidiogenous cells; **E–I**. Conidiophores, conidiogenous cells, and conidia; **J**. 1–2 septate conidia. Scale bars: 1 mm (**B**); 0.5 mm (**C**); 10 μm (**D–J**).

Palm plants are undoubtfully a good substrate for microfungal diversity due to their wide geographical distribution, high species richness, broad ecological range, and unique structural features. Multiple lines of evidence reveal that palm-associated fungal communities vary markedly among different tissue niches, such as roots, midribs and leaf blades. Furthermore, environmental conditions exert a stronger impact on the diversity and host specificity of these fungi than palm host identity or geographic location. (Hyde et al. 2000; Yanna and Hyde 2001, 2002; Liu 2014; Crous et al. 2024). Palm-associated fungi fulfill multifaceted ecological functions in tropical and subtropical ecosystems, functioning as saprobes decomposing dead palm litter, beneficial endophytes boosting host stress resistance, pathogenic taxa triggering leaf spot and rot diseases, and mycorrhizal symbionts facilitating nutrient uptake by palm roots (Pereira and Phillips 2023). Currently, research on fungal diversity associated with palms has attracted increasing attention from mycologists (Balslev et al. 2016; Baker and Dransfield 2016; Yao et al. 2023).

Yunnan Province in southwestern China stands out as a prominent global hotspot of fungal diversity, attributable to its unique low-latitude high-altitude topography, complex vertical climate gradients, well-preserved primitive tropical/subtropical vegetation, and long-term geological isolation that drives intensive co-evolution between plants and fungi (Hua et al. 2017; Feng and Yang 2018). In this study, two novel fungal species were isolated and characterized from palms in the Xishuangbanna Tropical Botanical Garden in southwestern China. The findings contribute to the known diversity of palm fungi and lay a foundation for future research on species diversity and diseases of palm plants.

## Supplementary Material

XML Treatment for Scolecobasidium yunnanense

XML Treatment for Yunnanomyces didymosporus

## Additional information

### Artificial Intelligence (AI) use

The authors accept full responsibility for the content of the manuscript, including the disclosure of any use of AI.

No AI tools were used in the preparation of this manuscript.

### Author ORCIDs

Q.-Y. Zhang https://orcid.org/0000-0001-9458-3566

Y.-H. Cao https://orcid.org/0009-0004-5072-3482

H. Sun https://orcid.org/0000-0003-2015-6136

L. Huang https://orcid.org/0000-0001-7536-0914

D.-W. Li https://orcid.org/0000-0002-2788-7938

L.-H. Zhu https://orcid.org/0000-0003-2740-4980

### Data availability

The new sequences are deposited in the GenBank database (Table 1).

## References

[B1] Abbott EV (1927) *Scolecobasidium*, a new genus of soil fungi. Mycologia 19: 29–31. 10.1080/00275514.1927.12020524

[B2] Arzanlou M, Groenewald JZ, Gams W, Braun U, Shin HD, Crous PW (2007) Phylogenetic and morphotaxonomic revision of *Ramichloridium* and allied genera. Studies in Mycology 58: 57–93. 10.3114/sim.2007.58.03 PMC2104745 18490996

[B3] Baker WJ, Dransfield J (2016) Beyond Genera Palmarum: Progress and prospects in palm systematics. Botanical Journal of the Linnean Society 182: 207–233. 10.1111/boj.12401

[B4] Balslev H, Bernal R, Fay MF (2016) Palms—emblems of tropical forests. Botanical Journal of the Linnean Society 182: 195–200. 10.1111/boj.12465

[B5] Barreto GG, Lima LMS, Castañeda-Ruiz RF, Costa-Rezende DH, Bezerra JDP, Gusmão LFP (2025) *Yunnanomyces syagri* sp. nov. (*Sympoventuriaceae*, *Venturiales*) on *Syagrus coronata*, an endemic Brazilian palm. Plant and Fungal Systematics 70: 65–74. 10.35535/pfsyst-2025-0007

[B6] Barron GL, Busch LV (1962) Studies on the soil hyphomycete *Scolecobasidium*. Canadian Journal of Botany 40: 77–84. 10.1139/b62-009

[B7] Crous PW, Carlier J, Roussel V, Groenewald JZ (2021) *Pseudocercospora* and allied genera associated with leaf spots of banana (*Musa* spp.). Fungal Systematics and Evolution 7: 1–19. 10.3114/fuse.2021.07.01 PMC8165963 34124615

[B8] Crous PW, Schumacher RK, Wingfield MJ, Akulov A, Denman S, Roux J, Braun U, Burgess TI, Carnegie AJ, Váczy KZ, Guatimosim E, Schwartsburd PB, Barreto RW, Hernández-Restrepo M, Lombard L, Groenewald JZ (2018) New and interesting fungi. 1. Fungal Systematics and Evolution 1: 169–215. 10.3114/fuse.2018.01.08 PMC7259438 32490366

[B9] Crous PW, Shivas RG, Quaedvlieg W, van der Bank M, Zhang Y, Summerell BA, Guarro J, Wingfield MJ, Wood AR, Alfenas AC, Braun U, Cano-Lira JF, García D, Marin-Felix Y, Alvarado P, Andrade JP, Armengol J, Assefa A, den Breeÿen A, Camele I, Cheewangkoon R, De Souza JT, Duong TA, Esteve-Raventós F, Fournier J, Frisullo S, García-Jiménez J, Gardiennet A, Gené J, Hernández-Restrepo M, Hirooka Y, Hospenthal DR, King A, Lechat C, Lombard L, Mang SM, Marbach PAS, Marincowitz S, Montaño-Mata NJ, Moreno G, Perez CA, Pérez Sierra AM, Robertson JL, Roux J, Rubio E, Schumacher RK, Stchigel AM, Sutton DA, Tan YP, Thompson EH, van der Linde E, Walker AK, Walker DM, Wickes BL, Wong PTW, Groenewald JZ (2014) Fungal Planet description sheets: 214–280. Persoonia 32: 184–306. 10.3767/003158514X682395 PMC4150077 25264390

[B10] Crous PW, Wingfield MJ, Burgess TI, Hardy GEStJ, Crane C, Barrett S, Cano-Lira JF, Le Roux JJ, Thangavel R, Guarro J, Stchigel AM, Martín MP, Alfredo DS, Barber PA, Barreto RW, Baseia IG, Cano-Canals J, Cheewangkoon R, Ferreira RJ, Gené J, Lechat C, Moreno G, Roets F, Shivas RG, Sousa JO, Tan YP, Wiederhold NP, Abell SE, Accioly T, Albizu JL, Alves JL, Antoniolli ZI, Aplin N, Araújo J, Arzanlou M, Bezerra JDP, Bouchara JP, Carlavilla JR, Castillo A, Castroagudín VL, Ceresini PC, Claridge GF, Coelho G, Coimbra VRM, Costa LA, Cunha KC, da Silva SS, Daniel R, de Beer ZW, Dueñas M, Edwards J, Enwistle P, Fiuza PO, Fournier J, García D, Gaitan A, Galán R, Gamboa P, Gil-Durán C, Girão M, Gomes AA, Gonçalves MFM, Gouliamova D, Groenewald JZ, Gusmão LFP, Haituk S, Heykoop M, Hirooka Y, Hofmann TA, Houbraken J, Hughes DP, Kautmanová I, Koppel O, Koukol O, Larsson E, Latha KPD, Lee DH, Lisboa DO, Lisboa WS, López-Villalba Á, Maciel JL, Manimohan P, Manjón JL, Marincowitz S, Marney TS, Meijer M, Miller AN, Olariaga I, Paiva LM, Piepenbring M, Poveda-Molero JC, Raj KNA, Raja HA, Rougeron A, Salcedo I, Samadi R, Santos T, Scarlett K, Seifert KA, Shuttleworth LA, Silva M, Silvério M, Sousa R, Storey R, Tanaka T, Taylor PWJ, Thines M, Torres JM, Vauras J, Verbruggen N, Vizzini A, Vos W, Yáñez-Morales MJ, Zima A (2016) Fungal Planet description sheets: 469–557. Persoonia 37: 218–403. 10.3767/003158516X694499 PMC5315290 28232766

[B11] Crous PW, Wingfield MJ, Jurjević Ž, Balashov S, Osieck ER, Marin-Felix Y, Luangsa-ard JJ, Mejía LC, Cappelli A, Parra LA, Lucchini G, Chen J, Moreno G, Faraoni M, Zhao RL, Weholt Ø, Borovička J, Jansen GM, Shivas RG, Tan YP, Akulov A, Alfenas AC, Alfenas RF, Altés A, Avchar R, Barreto RW, Catcheside DEA, Chi TY, Esteve-Raventós F, Fryar SC, Hanh LTMM, Larsbrink J, Oberlies NH, Olsson L, Pancorbo F, Raja HA, Thanh VN, Thuy NT, Ajithkumar K, Akram W, Alvarado P, Angeletti B, Arumugam E, Atashi Khalilabad A, Bandini D, Baroni TJ, Barreto GG, Boertmann D, Bose T, Castañeda Ruiz RF, Couceiro A, Cykowska-Marzencka B, Dai YC, Darmostuk V, da Silva SBG, Dearnaley JDW, Santiago ALCMA, Declercq B, Freitas LWS, De la Peña-Lastra S, Delgado G, de Lima CLF, Dhotre D, Dirks AC, Eisvand P, Erhard A, Ferro LO, García D, García-Martín A, Garrido-Benavent I, Gené J, Ghobad-Nejhad M, Gore G, Gunaseelan S, Gusmão LFP, Hammerbacher A, Hernández-Perez AT, Hernández-Restrepo M, Hofmann TA, Hubka V, Jiya N, Kaliyaperumal M, Keerthana KS, Ketabchi M, Kezo K, Knoppersen R, Kolarczyková D, Kumar TKA, Læssøe T, Langer E, Larsson E, Lodge DJ, Lynch MJ, Maciá-Vicente JG, Mahadevakumar S, Mateos A, Mehrabi-Koushki M, Miglio BV, Noor A, Oliveira JA, Pereira OL, Piątek M, Pinto A, Ramírez GH, Raphael B, Rawat G, Renuka M, Reschke K, Ruiz Mateo A, Saar I, Saba M, Safi A, Sánchez RM, Sandoval-Denis M, Savitha AS, Sharma A, Shelke D, Sonawane H, Souza MGAP, Stryjak-Bogacka M, Thines M, Thomas A, Torres-Garcia D, Traba JM, Vauras J, Vermaas M, Villarreal M, Vu D, Whiteside EJ, Zafari D, Starink-Willemse M, Groenewald JZ (2024) Fungal Planet description sheets: 1697–1780. Fungal Systematics and Evolution 14: 325–577. 10.3114/fuse.2024.14.19 PMC11736264 39830292

[B12] Crous PW, Wingfield MJ, Lombard L, Roets F, Swart WJ, Alvarado P, Carnegie AJ, Moreno G, Luangsa-ard JJ, Thangavel R, Alexandrova AV, Baseia IG, Bellanger JM, Bessette AE, Bessette AR, De la Peña-Lastra S, García D, Gené J, Pham THG, Heykoop M, Malysheva E, Malysheva V, Martín MP, Morozova OV, Noisripoom W, Overton BE, Rea AE, Sewall BJ, Smith ME, Smyth CW, Tasanathai K, Visagie CM, Adamčík S, Alves A, Andrade JP, Aninat MJ, Araújo RVB, Bordallo JJ, Boufleur T, Baroncelli R, Barreto RW, Bolin J, Cabero J, Caboň M, Cafà G, Caffot MLH, Cai L, Carlavilla JR, Chávez R, de Castro RRL, Delgat L, Deschuyteneer D, Dios MM, Domínguez LS, Evans HC, Eyssartier G, Ferreira BW, Figueiredo CN, Liu F, Fournier J, Galli-Terasawa LV, Gil-Durán C, Glienke C, Gonçalves MFM, Gryta H, Guarro J, Himaman W, Hywel-Jones N, Iturrieta-González I, Ivanushkina NE, Jargeat P, Khalid AN, Khan J, Kiran M, Kiss L, Kochkina GA, Kolařík M, Kubátová A, Lodge DJ, Loizides M, Luque D, Manjón JL, Marbach PAS, Massola Jr NS, Mata M, Miller AN, Mongkolsamrit S, Moreau PA, Morte A, Mostert L, Nadeem A, Nakashima C, Niskanen T, Nordén J, Nuñez M, Ochoa S, Olariaga I, Papp V, Pelaez F, Pérez-Vera R, Perrone G, Petrakova Z, Pildain MB, Pinruan U, Popov ES (2019) Fungal Planet description sheets: 951–1041. Persoonia 43: 223–425. 10.3767/persoonia.2019.43.06 PMC7085856 32214501

[B13] Dela Cruz TEE, Schulz BE, Kubicek CP, Druzhinina IS (2006) Carbon source utilization by the marine *Dendryphiella* species *D. arenaria* and *D. salina*. FEMS Microbiology Ecology 58: 343–353. 10.1111/j.1574-6941.2006.00184.x 17117979

[B14] Dransfield J, Uhl NW, Asmussen CB, Baker WJ, Harley MM, Lewis CE (2005) A new phylogenetic classification of the palm family, *Arecaceae*. Kew Bulletin 60: 559–569.

[B15] Dransfield J, Uhl NW, Asmussen CB, Baker WJ, Harley MM (2008) Genera Palmarum-the Evolution and Classification of the Palms. Royal Botanic Gardens, Kew, Richmond.

[B16] Feng B, Yang Z (2018) Studies on diversity of higher fungi in Yunnan, southwestern China: A review. Plant Diversity 40: 165–171. 10.1016/j.pld.2018.07.001 PMC6137262 30740561

[B17] Giraldo A, Sutton DA, Samerpitak K, De Hoog GS, Wiederhold NP, Guarro J, Gené J (2014) Occurrence of *Ochroconis* and *Verruconis* species in clinical specimens from the United States. Journal of Clinical Microbiology 52: 4189–4201. 10.1128/jcm.02027-14 PMC4313285 25232157

[B18] Glass NL, Donaldson GC (1995) Development of primer sets designed for use with the PCR to amplify conserved genes from filamentous ascomycetes. Applied and Environmental Microbiology 61: 1323–1330. 10.1128/aem.61.4.1323-1330.1995 PMC167388 7747954

[B19] Hall TA (1999) BioEdit: A user-friendly biological sequence alignment editor and analysis program for Windows 95/98/NT. Nucleic Acids Symposium Series 41(41): 95–98.

[B20] Hamayun M, Khan SA, Kim HY, Chaudhary MF, Kim IH, Lee IJ (2009) Gibberellin production and plant growth enhancement by newly isolated strain of *Scolecobasidium tshawytschae*. Journal of Microbiology and Biotechnology 19: 560–565. 10.4014/jmb.0809.520 19597313

[B21] Hao L, Chen C, Zhang R, Ma G, Wang M, Cao K, Sun G (2013) A new species of *Scolecobasidium* associated with the sooty blotch and flyspeck complex on banana from China. Mycological Progress 12: 489–495. 10.1007/s11557-012-0855-5

[B22] Hernández-Restrepo M, Giraldo A, Van Doorn R, Wingfield MJ, Groenewald JZ, Barreto RW, Colmán AA, Mansur PSC, Crous PW (2020) The Genera of Fungi–G6: *Arthrographis*, *Kramasamuha*, *Melnikomyces*, *Thysanorea*, and *Verruconis*. Fungal Systematics and Evolution 6: 1–24. 10.3114/fuse.2020.06.01 PMC7451779 32904189

[B23] Hibbett DS (1996) Phylogenetic evidence for horizontal transmission of group I introns in the nuclear ribosomal DNA of mushroom-forming fungi. Molecular Biology and Evolution 13: 903–917. 10.1093/oxfordjournals.molbev.a025658 8751999

[B24] Hua R, Chen Z, Fu W (2017) An overview of wild edible fungi resource conservation and its utilization in Yunnan. Journal of Agricultural Science 9: 158–169. 10.5539/jas.v9n5p158

[B25] Hyde KD, Taylor JE, Fröhlich J (2000) Genera of *Ascomycetes* from Palms. Fungal Diversity Press, Hong Kong.

[B26] Hyde KD, Noorabadi MT, Thiyagaraja V, He MQ, Johnston PR, Wijesinghe SN, Armand A, Biketova AY, Chethana KWT, Erdoğdu M, Ge ZW, Groenewald JZ, Hongsanan S, Kušan I, Leontyev DV, Li DW et al. (2024) The 2024 Outline of fungi-and fungus-like taxa. Mycosphere 15(1): 5146–6239. 10.5943/mycosphere/15/1/25

[B27] Jayasiri SC, Hyde KD, Jones EBG, McKenzie EHC, Jeewon R, Phillips AJL, Bhat DJ, Wanasinghe DN, Liu JK, Lu YZ, Kang JC, Xu J, Karunarathna SC (2019) Diversity, morphology and molecular phylogeny of *Dothideomycetes* on decaying wild seed pods and fruits. Mycosphere 10(1): 1–186. 10.5943/mycosphere/10/1/1

[B28] Johnston PR, Park D (2016) *Neocoleroa metrosideri* sp. nov. (*Sympoventuriaceae*, *Venturiales*). Phytotaxa 253: 214–218. 10.11646/phytotaxa.253.3.5

[B29] Kalyaanamoorthy S, Minh BQ, Wong TKF, von Haeseler A, Jermiin LS (2017) ModelFinder: Fast model selection for accurate phylogenetic estimates. Nature Methods 14: 587–589. 10.1038/nmeth.4285 PMC5453245 28481363

[B30] Karimi O, Hyde KD, Asghari R, Chethana KWT, Kaewchai S, Al-Otibi F, Li QR (2025) Peat swamp *Ascomycota* associated with palms (*Arecaceae*) from Narathiwat, Thailand. Mycosphere 16(1): 2456–2575. 10.5943/mycosphere/16/1/14

[B31] Katoh K, Rozewicki J, Yamada KD (2017) MAFFT online service: Multiple sequence alignment, interactive sequence choice and visualization. Briefings in Bioinformatics 20: 1160–1166. 10.1093/bib/bbx108 PMC6781576 28968734

[B32] Kornerup A, Wanscher JH (1978) Methuen Handbook of Color. Methuen and Co., Ltd., London, 252 pp.

[B33] Liu JK (2014) Phylogeny of *Ascomycetes* from Palms. PhD Thesis, Mae Fah Luang University, Chiang Rai, Thailand.

[B34] Liu NG, Hyde KD, Sun YR, Bhat DJ, Jones EBG, Jumpathong J, Lin CG, Lu YZ, Yang J, Liu JJ, Liu ZY, Liu JK (2024) Notes, outline, taxonomy and phylogeny of brown-spored hyphomycetes. Fungal Diversity 129: 1–281. 10.1007/s13225-024-00539-6

[B35] Liu YJ, Whelen S, Hall BD (1999) Phylogenetic relationships among ascomycetes: evidence from an RNA polymerase II subunit. Molecular Biology and Evolution 16: 1799–1808. 10.1093/oxfordjournals.molbev.a026092 10605121

[B36] Machouart M, Samerpitak K, De Hoog GS, Gueidan C (2014) A multigene phylogeny reveals that *Ochroconis* belongs to the family *Sympoventuriaceae* (*Venturiales*, *Dothideomycetes*). Fungal Diversity 65: 77–88. 10.1007/s13225-013-0252-7

[B37] Martin-Sanchez PM, Nováková A, Bastian F, Alabouvette C, Saiz-Jimenez C (2012) Two new species of the genus *Ochroconis*, *O. lascauxensi*s and *O. anomala* isolated from black stains in Lascaux Cave, France. Fungal Biology 116: 574–589. 10.1016/j.funbio.2012.02.006 22559918

[B38] Papan S, Preedanon S, Saengkaewsuk S, Klaysuban A, Kobmoo N, Pengsakun S, Yeemin T, Suetrong S, Sakayaroj J (2023) Genetic diversity of culturable fungi associated with scleractinian corals in the Gulf of Thailand. Botanica Marina 66: 309–318. 10.1515/bot-2022-0082

[B39] Peng W, Liu YJ, Wu N, Sun T, He XY, Gao YX, Wu CJ (2015) *Areca catechu* L. (*Arecaceae*): A review of its traditional uses, botany, phytochemistry, pharmacology and toxicology. Journal of Ethnopharmacology 164: 340–356. 10.1016/j.jep.2015.02.010 25681543

[B40] Pereira DS, Phillips AJL (2023) Palm fungi and their key role in biodiversity surveys: A review. Journal of Fungi 9: e1121. 10.3390/jof9111121 PMC10672035 37998926

[B41] Pratibha J, Prabhugaonkar A (2016) Distribution and phylogeny of *Mycosisymbrium cirrhosum*. Mycosphere 7: 44–50. 10.5943/mycosphere/7/1/5

[B42] Punithalingam E, Spooner BM (2011) A new fungicolous *Scolecobasidium (Hyphomycetes)* and *Caducirostrum* gen. nov. (coelomyces) from leaf litter in the UK and Italy. Kew Bulletin 66: 309–324. 10.1007/s12225-011-9289-5

[B43] Qiao M, Tian W, Castañeda-Ruiz RF, Xu J, Yu Z (2019) Two new species of *Verruconis* from Hainan, China. MycoKeys 48: 41–53. 10.3897/mycokeys.48.32147 PMC6414473 30872943

[B44] Ren J, Jie CY, Zhou QX, Li XH, Hyde KD, Jiang YL, Liu ZY (2013) Molecular and morphological data reveal two new species of *Scolecobasidium*. Mycoscience 54: 420–425. 10.1016/j.myc.2013.01.007

[B45] Ronquist F, Huelsenbeck JP (2003) MrBayes 3: Bayesian phylogenetic inference under mixed models. Bioinformatics 19: 1572–1574. 10.1093/bioinformatics/btg180 12912839

[B46] Roy RY, Dwivedi RS, Mishra RR (1962) Two new species of *Scolecobasidium* from soil. Lloydia 25: 164–166.

[B47] Safaie N, Salehi M, Felegari M, Farhadi S, Karimzadeh S, Asadi S, Yang JL, Naghavi MR (2024) Culture-based diversity of endophytic fungi of three species of *Ferula* grown in Iran. Frontiers in Microbiology 15: 1363158. 10.3389/fmicb.2024.1363158 PMC11153712 38846573

[B48] Samerpitak K, Van der Linde E, Choi HJ, Gerrits van den Ende AHG, Machouart M, Gueidan C, de Hoog GS (2013) Taxonomy of *Ochroconis*, genus including opportunistic pathogens on humans and animals. Fungal Diversity 65: 89–126. 10.1007/s13225-013-0253-6

[B49] Samerpitak K, van der Linde E, Choi HJ, Gerrits van den Ende B, Machouart M, Gueidan C, de Hoog GS (2014) Taxonomy of *Ochroconis*, genus including opportunistic pathogens on humans and animals. Fungal Diversity 65(1): 89–126. 10.1007/s13225-013-0253-6

[B50] Samerpitak K, Gerrits van den Ende AHG, Menken SBJ, De Hoog GS (2015) Three new species of the genus *Ochroconis*. Mycopathologia 180: 7–17. 10.1007/s11046-015-9910-5 PMC4498236 26093392

[B51] Samerpitak K, van den Ende BHG, Stielow JB, Menken SB, De Hoog GS (2016) Barcoding and species recognition of opportunistic pathogens in *Ochroconis* and *Verruconis*. Fungal Biology 120: 219–230. 10.1016/j.funbio.2015.08.010 26781378

[B52] Samerpitak K, Gloyna K, De Hoog GS (2017) A novel species of the oligotrophic genus *Ochroconis* colonizing indoor wet cells. Mycoscience 58: 290–296. 10.1016/j.myc.2017.04.002

[B53] Schoch CL, Robbertse B, Robert V, Vu D, Cardinali G, Irinyi L, Meyer W, Nilsson RH, Hughes K, Miller AN, Kirk PM, Abarenkov K, Aime MC, Ariyawansa HA, Bidartondo M, Boekhout T, Buyck B, Cai Q, Chen J, Crespo A, Crous PW, Damm U, de Beer ZW, Dentinger BTM, Divakar PK, Dueñas M, Feau N, Fliegerová K, García MA, Ge ZW, Griffith GW, Groenewald JZ, Groenewald M, Grube M, Gryzenhout M, Gueidan C, Guo L, Hambleton S, Hamelin R, Hansen K, Hofstetter V, Hong SB, Houbraken J, Hyde KD, Inderbitzin P, Johnston PR, Karunarathna SC, Kõljalg U, Kovács GM, Kraichak E, Krizsán K, Kurtzman CP, Larsson KH, Leavitt S, Letcher PM, Liimatainen K, Liu JK, Lodge DJ, Luangsa-ard JJ, Lumbsch HT, Maharachchikumbura SSN, Manamgoda D, Martin MP, Minnis AM, Moncalvo JM, Mule G, Nakasone KK, Niskanen T, Olariaga I, Papp T, Petkovits T, Pino-Bodas R, Powell MJ, Raja HA, Redecker D, Sarmiento-Ramírez JM, Seifert KA, Shrestha B, Stenroos S, Stielow B, Suh SO, Tanaka K, Tedersoo L, Tellería MT, Udayanga D, Untereiner WA, Uribeondo JD, Subbarao KV, Vágvölgyi C, Visagie C, Voigt K, Walker DM, Weir BS, Weiss M, Wijayawardene NN, Wingfield MJ, Xu JP, Yang ZL, Zhang N, Zhuang WY, Federhen S (2014) Finding needles in haystacks: Linking scientific names, reference specimens and molecular data for Fungi. Database 2014: bau061. 10.1093/database/bau061 PMC4075928 24980130

[B54] Seifert KA, Morgan-Jones G, Gams W, Kendrick B (2011) The genera of *Hyphomycetes*. CBS-KNAW Fungal Biodiversity Centre, Utrecht, The Netherlands.

[B55] Shen M, Zhang JQ, Zhao LL, Groenewald JZ, Crous PW, Zhang Y (2020) *Venturiales*. Studies in Mycology 96: 185–308. 10.1016/j.simyco.2020.03.001 PMC7452091 32904190

[B56] Song S, Li M, Huang JE, Liu F (2023) Two new species of *Scolecobasidium* (*Venturiales*, *Sympoventuriaceae*) associated with true mangrove plants and *S. terrestre* comb. nov. MycoKeys 96: 113–129. 10.3897/mycokeys.96.100621 PMC10210049 37252057

[B57] Stamatakis A (2014) RAxML Version 8: A tool for phylogenetic analysis and post-analysis of large phylogenies. Bioinformatics 30: 1312–1313. 10.1093/bioinformatics/btu033 PMC3998144 24451623

[B58] Suthar M, Singh SK, Singh PN (2025) *Scolecobasidium laurentii* (*Sympoventuriaceae*, *Venturiales*) a new species described based on morphology and molecular phylogeny from Western Ghats, India. Phytotaxa 711: 170–182. 10.11646/phytotaxa.711.2.7

[B59] Tibpromma S, Hyde KD, McKenzie EHC, Bhat DJ, Phillips AJL, Wanasinghe DN, Samarakoon MC, Jayawardena RS, Dissanayake AJ, Tennakoon DS, Doilom M, Phookamsak R, Tang AMC, Xu JC, Mortimer PE, Promputtha I, Maharachchikumbura SSN, Khan S, Karunarathna SC (2018) Fungal diversity notes 840–928: Micro-fungi associated with *Pandanaceae*. Fungal Diversity 93: 1–160. 10.1007/s13225-018-0408-6

[B60] Tsui CK, Berbee ML (2010) Transfer of two *Helicoma* species to *Troposporella* based on molecular and morphological data. Mycoscience 51: 144–148. 10.1007/s10267-009-0019-x

[B61] Verma IM (2014) Simplifying the direct submission process. Proceedings of the National Academy of Sciences 111: 14311–14311. 10.1073/pnas.1417688111 PMC4210033 25246596

[B62] Vilgalys R, Hester M (1990) Rapid genetic identification and mapping of enzymatically amplified ribosomal DNA from several *Cryptococcus* species. Journal of Bacteriology 172: 4238–4246. 10.1128/jb.172.8.4238-4246.1990 PMC213247 2376561

[B63] Vu D, Groenewald M, de Vries M, Gehrmann T, Stielow B, Eberhardt U, Al-Hatmi A, Groenewald JZ, Cardinali G, Houbraken J, Boekhout T, Crous PW, Robert V, Verkley GJM (2019) Large-scale generation and analysis of filamentous fungal DNA barcodes boosts coverage for kingdom fungi and reveals thresholds for fungal species and higher taxon delimitation. Studies in Mycology 92: 135–154. 10.1016/j.simyco.2018.05.001 PMC6020082 29955203

[B64] Wei TP, Zhang H, Zeng XY, Crous PW, Jiang YL (2022) Re-evaluation of *Sympoventuriaceae*. Persoonia 48: 219–260. 10.3767/persoonia.2022.48.07 PMC10792283 38234692

[B65] White TJ, Bruns T, Lee S, Taylor JW (1990) Amplification and direct sequencing of fungal ribosomal RNA genes for phylogenetics. In: Innis MA, Gelfand DH, Sninsky JJ, White TJ (Eds) PCR protocols: A guide to methods and applications. Academic Press, New York, 315–322. 10.1016/B978-0-12-372180-8.50042-1

[B66] Yanna WHH, Hyde KD (2001) Fungal communities on decaying palm fronds in Australia, Brunei, and Hong Kong. Mycological Research 105: 1458–1471. 10.1017/S0953756201005214

[B67] Yanna WHH, Hyde KD (2002) Fungal succession on fronds of *Phoenix hanceana* in Hong Kong. Fungal Diversity 10: 185–211.

[B68] Yao G, Zhang YQ, Barrett C, Xue B, Bellot S, Baker WJ, Ge XJ (2023) A plastid phylogenomic framework for the palm family (*Arecaceae*). BMC Biology 21: 50. 10.1186/s12915-023-01544-y PMC9993706 36882831

[B69] Yarita K, Sano A, Samerpitak K, Kamei K, de Hoog GS, Nishimura K (2010) *Ochroconis calidifluminalis*, a sibling of the neurotropic pathogen *O. gallopava*, isolated from hot spring. Mycopathologia 170: 21–30. 10.1007/s11046-010-9292-7 20213501

[B70] Zalar P, Gubenšek A, Gostincar C, Kostanjšek R, Bizjak-Mali L, Gunde-Cimerman N (2022) Cultivable skin mycobiota of healthy and diseased blind cave salamander (*Proteus anguinus*). Frontiers in Microbiology 13: e926558. 10.3389/fmicb.2022.926558 PMC9329069 35910647

[B71] Zhang D, Gao F, Jakovlić I, Zou H, Zhang J, Li WX, Wang GT (2020) PhyloSuite: An integrated and scalable desktop platform for streamlined molecular sequence data management and evolutionary phylogenetics studies. Molecular Ecology Resources 20: 348–355. 10.1111/1755-0998.13096 31599058

[B72] Zhang QY, Huang JH, Ren JL, Zhang Y, Zhao CL, Si J (2026) Morphological and phylogenetic analyses reveal a new genus and two new species of *Hymenochaetales (Basidiomycota)* from southeast China. MycoKeys 127: 25–42. 10.3897/mycokeys.127.171179 PMC12800780 41542017

[B73] Zhang SN, Liu JK, Jones EBG, Cheewangkoon R (2019) Morphology and phylogeny of *Yunnanomyces phoenicis* sp. nov. (*Sympoventuriaceae*) from Thailand. Phytotaxa 409: 223–234. 10.5943/ajom/2/1/12

[B74] Zhang SN, Hyde KD, Jones EBG, Yu YD, Cheewangkoon R, Liu JK (2024a) Current insights into palm fungi with emphasis on taxonomy and phylogeny. Fungal Diversity 127: 55–301. 10.1007/s13225-024-00536-9

[B75] Zhang ZY, Pan H, Tao G, Li X, Han YF, Feng Y, Tong SQ, Ding CY (2024b) Culturable mycobiota from Guizhou Wildlife Park in China. Mycosphere 15: 654–763. 10.5943/mycosphere/15/1/5

[B76] Zhou L, Zhang QY, Wan Y, Chen YL, Li DW, Tan YH, Zhu LH (2025) Three novel species of *Cladosporium* and *Sarocladium* isolated from palm trees. MycoKeys 123: 29–51. 10.3897/mycokeys.123.165471 PMC12489491 41048881

